# A Hybrid Classification System for Heart Disease Diagnosis Based on the RFRS Method

**DOI:** 10.1155/2017/8272091

**Published:** 2017-01-03

**Authors:** Xiao Liu, Xiaoli Wang, Qiang Su, Mo Zhang, Yanhong Zhu, Qiugen Wang, Qian Wang

**Affiliations:** ^1^School of Economics and Management, Tongji University, Shanghai, China; ^2^School of Economics and Management, Shanghai Maritime University, Shanghai, China; ^3^Department of Scientific Research, Shanghai General Hospital, School of Medicine, Shanghai Jiaotong University, Shanghai, China; ^4^Trauma Center, Shanghai General Hospital, School of Medicine, Shanghai Jiaotong University, Shanghai, China

## Abstract

Heart disease is one of the most common diseases in the world. The objective of this study is to aid the diagnosis of heart disease using a hybrid classification system based on the ReliefF and Rough Set (RFRS) method. The proposed system contains two subsystems: the RFRS feature selection system and a classification system with an ensemble classifier. The first system includes three stages: (i) data discretization, (ii) feature extraction using the ReliefF algorithm, and (iii) feature reduction using the heuristic Rough Set reduction algorithm that we developed. In the second system, an ensemble classifier is proposed based on the C4.5 classifier. The Statlog (Heart) dataset, obtained from the UCI database, was used for experiments. A maximum classification accuracy of 92.59% was achieved according to a jackknife cross-validation scheme. The results demonstrate that the performance of the proposed system is superior to the performances of previously reported classification techniques.

## 1. Introduction

Cardiovascular disease (CVD) is a primary cause of death. An estimated 17.5 million people died from CVD in 2012, representing 31% of all global deaths (http://www.who.int/mediacentre/factsheets/fs317/en/). In the United States, heart disease kills one person every 34 seconds [1].

Numerous factors are involved in the diagnosis of heart disease, which complicates a physician's task. To help physicians make quick decisions and minimize errors in diagnosis, classification systems enable physicians to rapidly examine medical data in considerable detail [2]. These systems are implemented by developing a model that can classify existing records using sample data. Various classification algorithms have been developed and used as classifiers to assist doctors in diagnosing heart disease patients.

The performances obtained using the Statlog (Heart) dataset [3] from the UCI machine learning database are compared in this context. Lee [4] proposed a novel supervised feature selection method based on the bounded sum of weighted fuzzy membership functions (BSWFM) and Euclidean distances and obtained an accuracy of 87.4%. Tomar and Agarwal [5] used the* F*-score feature selection method and the Least Square Twin Support Vector Machine (LSTSVM) to diagnose heart diseases, obtaining an average classification accuracy of 85.59%. Buscema et al. [6] used the Training with Input Selection and Testing (TWIST) algorithm to classify patterns, obtaining an accuracy of 84.14%. The Extreme Learning Machine (ELM) has also been used as a classifier, obtaining a reported classification accuracy of 87.5% [7]. The genetic algorithm with the Naïve Bayes classifier has been shown to have a classification accuracy of 85.87% [8]. Srinivas et al. [9] obtained an 83.70% classification accuracy using Naïve Bayes. Polat and Güneş [10] used the RBF kernel* F*-score feature selection method to detect heart disease. The LS-SVM classifier was used, obtaining a classification accuracy of 83.70%. In [11], the GA-AWAIS method was used for heart disease detection, with a classification accuracy of 87.43%. The Algebraic Sigmoid Method has also been proposed to classify heart disease, with a reported accuracy of 85.24% [12]. Wang et al. [13] used linear kernel SVM classifiers for heart disease detection and obtained an accuracy of 83.37%. In [14], three distance criteria were applied in simple AIS, and the accuracy obtained on the Statlog (Heart) dataset was 83.95%. In [15], a hybrid neural network method was proposed, and the reported accuracy was 86.8%. Yan et al. [16] achieved an 83.75% classification accuracy using ICA and SVM classifiers. Şahan et al. [17] proposed a new artificial immune system named the Attribute Weighted Artificial Immune System (AWAIS) and obtained an accuracy of 82.59% using the* k*-fold cross-validation method. In [18], the* k*-NN,* k*-NN with Manhattan, feature space mapping (FSM), and separability split value (SSV) algorithms were used for heart disease detection, and the highest classification accuracy (85.6%) was obtained by* k*-NN.

From these works, it can be observed that feature selection methods can effectively increase the performance of single classifier algorithms in diagnosing heart disease [19]. Noisy features and dependency relationships in the heart disease dataset can influence the diagnosis process. Typically, there are numerous records of accompanied syndromes in the original datasets as well as a large number of redundant symptoms. Consequently, it is necessary to reduce the dimensions of the original feature set by a feature selection method that can remove the irrelevant and redundant features.

ReliefF is one of the most popular and successful feature estimation algorithms. It can accurately estimate the quality of features with strong dependencies and is not affected by their relations [20]. There are two advantages to using the ReliefF algorithm: (i) it follows the filter approach and does not employ domain specific knowledge to set feature weights [21, 22], and (ii) it is a feature weighting (FW) engineering technique. ReliefF assigns a weight to each feature that represents the usefulness of that feature for distinguishing pattern classes. First, the weight vector can be used to improve the performance of the lazy algorithms [21]. Furthermore, the weight vector can also be used as a method for ranking features to guide the search for the best subset of features [22–26]. The ReliefF algorithm has proved its usefulness in FS [20, 23], feature ranking [27], and building tree-based models [22], with an association rules-based classifier [28], in improving the efficiencies of the genetic algorithms [29] and with lazy classifiers [21].

ReliefF has excellent performance in both supervised and unsupervised learning. However, it does not help identify redundant features [30–32]. ReliefF algorithm estimates the quality of each feature according to its weight. When most of the given features are relevant to the concept, this algorithm will select most of them even though only some fraction is necessary for concept description [32]. Furthermore, the ReliefF algorithm does not attempt to determine the useful subsets of these weakly relevant features [33].

Redundant features increase dimensionality unnecessarily [34] and adversely affect learning performance when faced with shortage of data. It has also been empirically shown that removing redundant features can result in significant performance improvement [35]. Rough Set (RS) theory is a new mathematical approach to data analysis and data mining that has been applied successfully to many real-life problems in medicine, pharmacology, engineering, banking, financial and market analysis, and others [36]. The RS reduction algorithm can reduce all redundant features of datasets and seek the minimum subset of features to attain a satisfactory classification [37].

There are three advantages to combining ReliefF and RS (RFRS) approach as an integrated feature selection system for heart disease diagnosis.

(i) The RFRS method can remove superfluous and redundant features more effectively. The ReliefF algorithm can select relevant features for disease diagnosis; however, redundant features may still exist in the selected relevant features. In such cases, the RS reduction algorithm can remove remaining redundant features to offset this limitation of the ReliefF algorithm.

(ii) The RFRS method helps to accelerate the RS reduction process and guide the search of the reducts. Finding a minimal reduct of a given information system is an NP-hard problem, as was demonstrated in [38]. The complexity of computing all reducts in an information system is rather high [39]. On one hand, as a data preprocessing tool, the features revealed by the ReliefF method can accelerate the operation process by serving as the input for the RS reduction algorithm. On the other hand, the weight vector obtained by the ReliefF algorithm can act as a heuristic to guide the search for the reducts [25, 26], thus helping to improve the performance of the heuristic algorithm [21].

(iii) The RFRS method can reduce the number and improve the quality of reducts. Usually, more than one reduct exists in the dataset; and larger numbers of features result in larger numbers of reducts [40]. The number of reducts will decrease if superfluous features are removed using the ReliefF algorithm. When unnecessary features are removed, more important features can be extracted, which will also improve the quality of reducts.

It is obvious that the choice of an efficient feature selection method and an excellent classifier is extremely important for the heart disease diagnosis problem [41]. Most of the common classifiers from the machine learning community have been used for heart disease diagnosis. It is now recognized that no single model exists that is superior for all pattern recognition problems, and no single technique is applicable to all problems [42]. One solution to overcome the limitations of a single classifier is to use an ensemble model. An ensemble model is a multiclassifier combination model that results in more precise decisions because the same problem is solved by several different trained classifiers, which reduces the variance of error estimation [43]. In recent years, ensemble learning has been employed to increase classification accuracies beyond the level that can be achieved by individual classifiers [44, 45]. In this paper, we used an ensemble classifier to evaluate the feature selection model.

To improve the efficiency and effectiveness of the classification performance for the diagnosis of heart disease, we propose a hybrid classification system based on the ReliefF and RS (RFRS) approach in handling relevant and redundant features. The system contains two subsystems: the RFRS feature selection subsystem and a classification subsystem. In the RFRS feature selection subsystem, we use a two-stage hybrid modeling procedure by integrating ReliefF with the RS (RFRS) method. First, the proposed method adopts the ReliefF algorithm to obtain feature weights and select more relevant and important features from heart disease datasets. Then, the feature estimation obtained from the first phase is used as the input for the RS reduction algorithm and guide the initialization of the necessary parameters for the genetic algorithm. We use a GA-based search engine to find satisfactory reducts. In the classification subsystem, the resulting reducts serve as the input for the chosen classifiers. Finally, the optimal reduct and performance can be obtained.

To evaluate the performance of the proposed hybrid method, a confusion matrix, sensitivity, specificity, accuracy, and ROC were used. The experimental results show that the proposed method achieves very promising results using the jack knife test.

The main contributions of this paper are summarized as follows.

(i) We propose a feature selection system to integrate the ReliefF approach with the RS method (RFRS) to detect heart disease in an efficient and effective way. The idea is to use the feature estimation from the ReliefF phase as the input and heuristics for the RS reduction phase.

(ii) In the classification system, we propose an ensemble classifier using C4.5 as the base classifier. Ensemble learning can achieve better performance at the cost of computation than single classifiers. The experimental results show that the ensemble classifier in this paper is superior to three common classifiers.

(iii) Compared with three classifiers and previous studies, the proposed diagnostic system achieved excellent classification results. On the Statlog (Heart) dataset from the UCI machine learning database [3], the resulting classification accuracy was 92.59%, which is higher than that achieved by other studies.

The rest of the paper is organized as follows.  Section 2 offers brief background information concerning the ReliefF algorithm and RS theory. The details of the diagnosis system implementation are presented in Section 3.  Section 4 describes the experimental results and discusses the proposed method. Finally, conclusions and recommendations for future work are summarized in Section 5.

## 2. Theoretical Background

### 2.1. Basic Concepts of Rough Set Theory

Rough Set (RS) theory, which was proposed by Pawlak, in the early 1980s, is a new mathematical approach to addressing vagueness and uncertainty [46]. RS theory has been applied in many domains, including classification system analysis, pattern reorganization, and data mining [47]. RS-based classification algorithms are based on equivalence relations and have been used as classifiers in medical diagnosis [37, 46]. In this paper, we primarily focus on the RS reduction algorithm, which can reduce all redundant features of datasets and seek the minimum subset of features necessary to attain a satisfactory classification [37]. A few basic concepts of RS theory are defined [46, 47] as follows.

**Definition 1 .**

*U* is a certain set that is referred to as the universe;* R* is an equivalence relation in* U*. The pair *A* = (*U*, *R*) is referred to as an approximation space.

**Definition 2 .**

*P* ⊂ *R*, ∩*P* (the intersection of all equivalence relations in* P*) is an equivalence relation, which is referred to as the* R*-indiscernibility relation, and it is represented by Ind⁡(*R*).

**Definition 3 .**

Let* X* be a certain subset of* U*. The least composed set in* R* that contains* X* is referred to as the best upper approximation of* X* in* R* and represented by *R* ^−^(*X*); the greatest composed set in* R* contained in* X* is referred to as the best lower approximation of* X* in* R*, and it is represented by *R*_−_(*X*).

(1) R−X=x∈U:xR⊂X,R−X=x∈U:xR∩X≠ϕ.

**Definition 4 .**

An information system is denoted as

(2) $$\begin{array}{c} S=\left(\;U,A,V,F\;\right), \end{array}$$

where* U* is the universe that consists of a finite set of* n* objects, *A* = {*C* ∪ *D*}, in which* C* is a set of condition attributes and* D* is a set of decision attributes,* V* is the set of domains of attributes, and* F* is the information function for each *a* ∈ *A*, *x* ∈ *U*, *F*(*x*, *a*) ∈ *V*_*a*_.

**Definition 5 .**

In an information system,* C* and* D* are sets of attributes in *U*. *X* ∈ *U*/ind⁡(*Q*), and pos_*p*_(*Q*), which is referred to as a positive region, is defined as

(3) $$\begin{array}{c} {\text{pos}}_{p}\left(\;Q\;\right)=\cup P_{-}\left(\;X\;\right). \end{array}$$

**Definition 6 .**

*P* and* Q* are sets of attributes in* U*, *P*, *Q*⊆*A*, and the dependency *r*_*p*_(*Q*) is defined as

(4) $$\begin{array}{c} r_{p}\left(\;Q\;\right)=\frac{card\left({\text{pos}}_{p}\left(Q\right)\right)}{card\left(U\right)}. \end{array}$$

Card (*X*) denotes the cardinality of* X*. 0 ≤ *r*_*p*_(*Q*) ≤ 1.

**Definition 7 .**

*P* and* Q* are sets of attributes in* U*, *P*, *Q*⊆*A*, and the significance of *a*_*i*_ is defined as

(5) $$\begin{array}{c} \mathrm{sig}\left(\;\left\{\;a_{i}\;\right\}\;\right)=r_{p}\left(\;Q\;\right)-r_{p-a_{i}}\left(\;Q\;\right). \end{array}$$

### 2.2. ReliefF Algorithm

Many feature selection algorithms have been developed; ReliefF is one of the most widely used and effective algorithms [48]. ReliefF is a simple yet efficient procedure for estimating the quality of features in problems with dependencies between features [20]. The pseudocode of ReliefF algorithm is listed in Algorithm 1.

## 3. Proposed System

### 3.1. Overview

**Figure 1:**
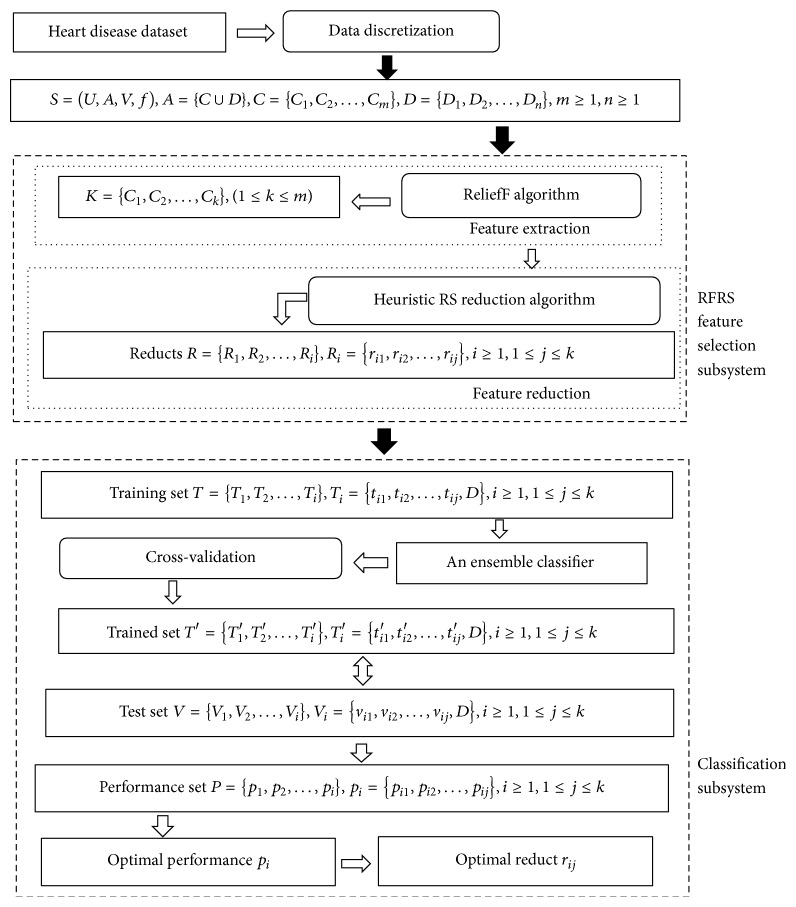
Structure of RFRS-based classification system.

The proposed hybrid classification system consists of two main components: (i) feature selection using the RFRS subsystem and (ii) data classification using the classification system. A flow chart of the proposed system is shown in Figure 1. We describe the preprocessing and classification systems in the following subsections.

### 3.2. RFRS Feature Selection Subsystem

We propose a two-phase feature selection method based on the ReliefF algorithm and the RS (RFRS) algorithm. The idea is to use the feature estimation from the ReliefF phase as the input and heuristics for the subsequent RS reduction phase. In the first phase, we adopt the ReliefF algorithm to obtain feature weights and select important features; in the second phase, the feature estimation obtained from the first phase is used to guide the initialization of the parameters required for the genetic algorithm. We use a GA-based search engine to find satisfactory reducts.

The RFRS feature selection subsystem consists of three main modules: (i) data discretization, (ii) feature extraction using the ReliefF algorithm, and (iii) feature reduction using the heuristic RS reduction algorithm we propose.

#### 3.2.1. Data Discretization

RS reduction requires categorical data. Consequently, data discretization is the first step. We used an approximate equal interval binning method to bin the data variables into a small number of categories.

#### 3.2.2. Feature Extraction by the ReliefF Algorithm

Module 2 is used for feature extraction by the ReliefF algorithm. To deal with incomplete data, we change the diff function. Missing feature values are treated probabilistically [20]. We calculate the probability that two given instances have different values for a given feature conditioned over the class value [20]. When one instance has an unknown value, then

(6) $$\begin{array}{c} \text{diff}\left(\;A,I_{1},I_{2}\;\right)=1-P\left(\;\text{value}\left(\;A,I_{2}\;\right)\;|\;\text{class}\left(\;I_{1}\;\right)\;\right). \end{array}$$

When both instances have unknown values, then

(7) diffA,I1,I2=1−∑V#valuesAPV ∣ classI1×PV ∣ classI2.

Conditional probabilities are approximated by relative frequencies in the training set. The process of feature extraction is shown as follows.

*The Process of Feature Extraction Using ReliefF Algorithm*

*Input*. A decision table *S* = (*U*, *P*, *Q*), *P* = {*a*_1_, *a*_2_,…, *a*_*m*_}, *Q* = {*d*_1_, *d*_2_,…, *d*_*n*_} (*m* ≥ 1, *n* ≥ 1).

*Output*. The selected feature subset *K* = {*a*_1_, *a*_2_,…, *a*_*k*_}(1 ≤ *k* ≤ *m*).

*Step  1*. Obtain the weight matrix of each feature using ReliefF algorithm*W* = {*w*_1_, *w*_2_,…, *w*_*i*_,…, *w*_*m*_}  (1 ≤ *i* ≤ *m*).

*Step  2*. Set a threshold, *δ*. 

*Step  3*. If *w*_*i*_ > *δ*, then feature *a*_*i*_ is selected.

#### 3.2.3. Feature Reduction by the Heuristic RS Reduction Algorithm

The evaluation result obtained by the ReliefF algorithm is the feature rank. A higher ranking means that the feature has stronger distinguishing qualities and a higher weight [30]. Consequently, in the process of reduct searching, the features in the front rank should have a higher probability of being selected.

We proposed the RS reduction algorithm by using the feature estimation as heuristics and a GA-based search engine to search for the satisfactory reducts. The pseudocode of the algorithm is provided in Algorithm 2. The algorithm was implemented in MATLAB R2014a.

### 3.3. Classification Subsystem

In the classification subsystem, the dataset is split into training sets and corresponding test sets. The decision tree is a nonparametric learning algorithm that does not need to search for optimal parameters in the training stage and thus is used as a weak learner for ensemble learning [49]. In this paper, the ensemble classifier uses the C4.5 decision tree as the base classifier. We use the boosting technique to construct ensemble classifiers. Jackknife cross-validation is used to increase the amount of data for testing the results. The optimal reduct is the reduct that obtains the best classification accuracy.

## 4. Experimental Results

### 4.1. Dataset

The Statlog (Heart) dataset used in our work was obtained from the UCI machine learning database [3]. This dataset contains 270 observations and 2 classes: the presence and absence of heart disease. The samples include 13 condition features, presented in Table 1. We denote the 13 features as* C*_1_ to* C*_13_.

### 4.2. Performance Evaluation Methods

#### 4.2.1. Confusion Matrix, Sensitivity, Specificity, and Accuracy

A confusion matrix [50] contains information about actual and predicted classifications performed by a classification system. The performance of such systems is commonly evaluated using the data in the matrix.  Table 2 shows the confusion matrix for a two-class classifier.

In the confusion matrix, TP is the number of true positives, representing the cases with heart disease that are correctly classified into the heart disease class. FN is the number of false negatives, representing cases with heart disease that are classified into the healthy class. TN is the number of true negatives, representing healthy cases that are correctly classified into the healthy class. Finally, FP is the number of false positives, representing the healthy cases that are incorrectly classified into the heart disease class [50].

The performance of the proposed system was evaluated based on sensitivity, specificity, and accuracy tests, which use the true positive (TP), true negative (TN), false negative (FN), and false positive (FP) terms [33]. These criteria are calculated as follows [41]:

(8) SensitivitySn=TPTP+FN×100%,SpecificitySp=TNFP+TN×100%,AccuracyAcc=TP+TNTP+TN+FP+FN×100%.

#### 4.2.2. Cross-Validation

Three cross-validation methods, namely, subsampling tests, independent dataset tests, and jackknife tests, are often employed to evaluate the predictive capability of a predictor [51]. Among the three methods, the jackknife test is deemed the least arbitrary and the most objective and rigorous [52, 53] because it always yields a unique outcome, as demonstrated by a penetrating analysis in a recent comprehensive review [54, 55]. Therefore, the jackknife test has been widely and increasingly adopted in many areas [56, 57].

Accordingly, the jackknife test was employed to examine the performance of the model proposed in this paper. For jackknife cross-validation, each sequence in the training dataset is, in turn, singled out as an independent test sample and all the parameter rules are calculated based on the remaining samples, without including the one being treated as the test sample.

#### 4.2.3. Receiver Operating Characteristics (ROC)

The receiver operating characteristic (ROC) curve is used for analyzing the prediction performance of a predictor [58]. It is usually plotted using the true positive rate versus the false positive rate, as the discrimination threshold of classification algorithm is varied. The area under the ROC curve (AUC) is widely used and relatively accepted in classification studies because it provides a good summary of a classifier's performance [59].

### 4.3. Results and Discussion

#### 4.3.1. Results and Analysis on the Statlog (Heart) Dataset

First, we used the equal interval binning method to discretize the original data. In the feature extraction module, the number of* k*-nearest neighbors in the ReliefF algorithm was set to 10, and the threshold, *δ*, was set to 0.02.  Table 3 summarizes the results of the ReliefF algorithm. Based on these results,* C*_5_ and* C*_6_ were removed. In Module 3, we obtained 15 reducts using the heuristic RS reduction algorithm implemented in MATLAB 2014a.

Trials were conducted using 70%–30% training-test partitions, using all the reduced feature sets. Jackknife cross-validation was performed on the dataset. The number of desired base classifiers* k* was set to 50, 100, and 150. The calculations were run 10 times, and the highest classification performances for each training-test partition are provided in Table 4.

**Figure 2:**
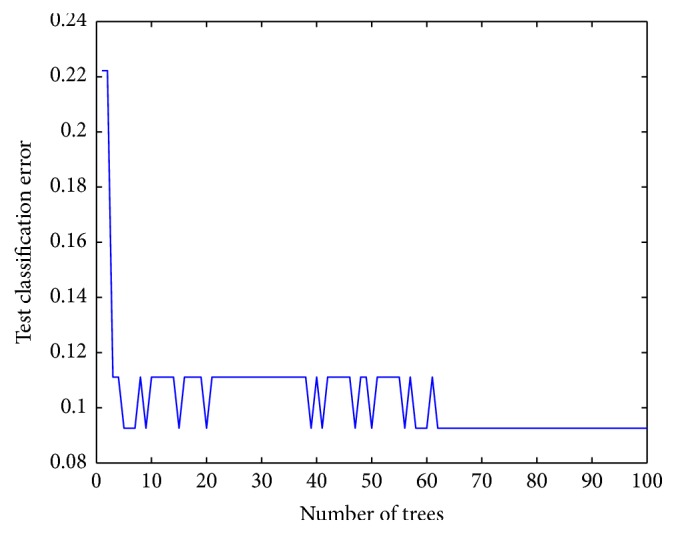
Training process of *R*_7_.

**Figure 3:**
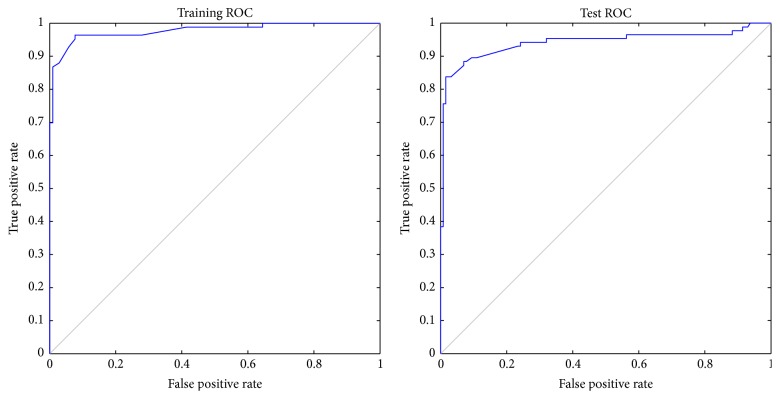
ROC curves for training and test sets.

In Table 4, *R*_2_ obtains the best test set classification accuracy (92.59%) using the ensemble classifiers when *k* = 100. The training process is shown in Figure 2. The training and test ROC curves are shown in Figure 3.

#### 4.3.2. Comparison with Other Classifiers

In this section, our ensemble classification method is compared with the individual C4.5 decision tree and Naïve Bayes and Bayesian Neural Networks (BNN) methods. The C4.5 decision tree and Naïve Bayes are common classifiers. Bayesian Neural Networks (BNN) is a classifier that uses Bayesian regularization to train feed-forward neural networks [60] and has better performance than pure neural networks. The classification accuracy results of the four classifiers are listed in Table 5. The ensemble classification method has better performance than the individual C4.5 classifier and the other two classifiers.

#### 4.3.3. Comparison of the Results with Other Studies

We compared our results with the results of other studies.  Table 6 shows the classification accuracies of our study and previous methods.

The results show that our proposed method obtains superior and promising results in classifying heart disease patients. We believe that the proposed RFRS-based classification system can be exceedingly beneficial in assisting physicians in making accurate decisions.

## 5. Conclusions and Future Work

In this paper, a novel ReliefF and Rough Set- (RFRS-) based classification system is proposed for heart disease diagnosis. The main novelty of this paper lies in the proposed approach: the combination of the ReliefF and RS methods to classify heart disease problems in an efficient and fast manner. The RFRS classification system consists of two subsystems: the RFRS feature selection subsystem and the classification subsystem. The Statlog (Heart) dataset from the UCI machine learning database [3] was selected to test the system. The experimental results show that the reduct *R*_2_ (*C*_1_, *C*_3_, *C*_7_, *C*_8_, *C*_11_, *C*_12_, *C*_13_) achieves the highest classification accuracy (92.59%) using an ensemble classifier with the C4.5 decision tree as the weak learner. The results also show that the RFRS method has superior performance compared to three common classifiers in terms of ACC, sensitivity, and specificity. In addition, the performance of the proposed system is superior to that of existing methods in the literature. Based on empirical analysis, the results indicate that the proposed classification system can be used as a promising alternative tool in medical decision making for heart disease diagnosis.

However, the proposed method also has some weaknesses. The number of the nearest neighbors (*k*) and the weight threshold (*θ*) are not stable in the ReliefF algorithm [20]. One solution to this problem is to compute estimates for all possible numbers and take the highest estimate of each feature as the final result [20]. We need to perform more experiments to find the optimal parameter values for the ReliefF algorithm in the future.

## Figures and Tables

**Algorithm 1:**
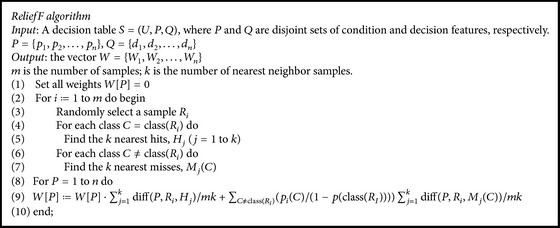
Pseudocode of ReliefF.

**Algorithm 2:**
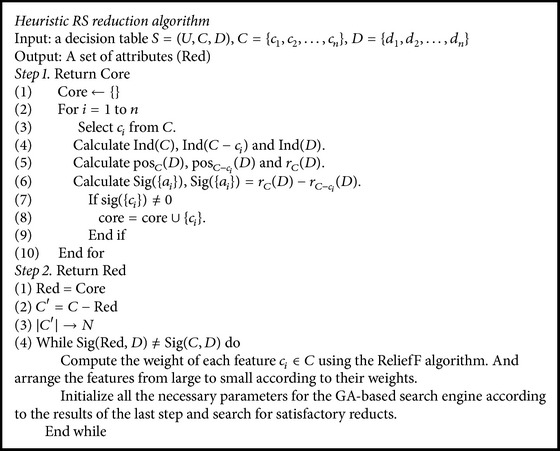
Pseudocode of heuristic RS reduction algorithm.

**Table 1:** Feature information of Statlog (Heart) dataset.

Feature	Code	Description	Domain	Data type	Mean	Standard deviation
Age	*C*_1_	—	29–77	Real	54	9
Sex	*C*_2_	Male, female	0, 1	Binary	—	—
Chest pain type	*C*_3_	Angina, asymptomatic, abnormal	1, 2, 3, 4	Nominal	—	—
Resting blood pressure	*C*_4_	—	94–200	Real	131.344	17.862
Serum cholesterol in mg/dl	*C*_5_	—	126–564	Real	249.659	51.686
Fasting blood sugar > 120 mg/dl	*C*_6_	—	0, 1	Binary	—	—
Resting electrocardiographic results	*C*_7_	Norm, abnormal, hyper	0, 1, 2	Nominal	—	—
Maximum heart rate achieved	*C*_8_	—	71–202	Real	149.678	23.1666
Exercise-induced angina	*C*_9_	—	0, 1	Binary	—	—
Old peak = ST depression induced by exercise relative to rest	*C*_10_	—	0–6.2	Real	1.05	1.145
Slope of the peak exercise ST segment	*C*_11_	Up, flat, down	1, 2, 3	Ordered	—	—
Number of major vessels (0–3) colored by fluoroscopy	*C*_12_	—	0, 1, 2, 3	Real	—	—
Thal	*C*_13_	Normal, fixed defect, reversible defect	3, 6, 7	Nominal	—	—

**Table 2:** The confusion matrix.

	Predicted patients with heart disease	Predicted healthy persons
Actual patients with heart disease	True positive (TP)	False negative (FN)
Actual healthy persons	False positive (FP)	True negative (TN)

**Table 3:** Results of the ReliefF algorithm.

Feature	*C*_2_	*C*_13_	*C*_7_	*C*_12_	*C*_9_	*C*_3_	*C*_11_	*C*_10_	*C*_8_	*C*_4_	*C*_1_	*C*_6_	*C*_5_
Weight	0.172	0.147	0.126	0.122	0.106	0.098	0.057	0.046	0.042	0.032	0.028	0.014	0.011

**Table 4:** Performance values for different reduced subset.

Code	Reduct	Number	Test classification accuracy (%)			
			Ensemble classifier			
			*K*	Sn	Sp	ACC
*R*_1_	*C*_3_, *C*_4_, *C*_7_, *C*_8_, *C*_10_, *C*_12_, *C*_13_	7	50	83.33	87.5	85.19
			100	83.33	95.83	88.89
			150	86.67	83.33	85.19
*R*_2_	*C*_1_, *C*_3_, *C*_7_, *C*_8_, *C*_11_, *C*_12_, *C*_13_	7	50	86.67	91.67	88.89
			100	93.33	87.50	92.59
			150	93.33	87.04	90.74
*R*_3_	*C*_1_, *C*_2_, *C*_4_, *C*_7_, *C*_8_, *C*_9_, *C*_12_	7	50	86.67	83.33	85.19
			100	93.33	79.17	87.04
			150	80	91.67	85.19
*R*_4_	*C*_1_, *C*_4_, *C*_7_, *C*_8_, *C*_10_, *C*_11_, *C*_12_, *C*_13_	8	50	86.67	83.33	85.19
			100	93.33	83.33	88.89
			150	86.67	87.5	87.04

**Table 5:** Classification results using the four classifiers.

Classifiers	Test classification accuracy of *R*_2_ (%)		
	Sn	Sp	Acc
Ensemble classifier (*k* = 50)	86.67	91.67	88.89
Ensemble classifier (*k* = 100)	93.33	87.50	92.59
Ensemble classifier (*k* = 150)	93.33	87.04	90.74
C4.5 tree	93.1	80	87.03
Naïve Bayes	93.75	68.18	83.33
Bayesian Neural Networks (BNN)	93.75	72.72	85.19

**Table 6:** Comparison of our results with those of other studies.

Author	Method	Classification accuracy (%)
Our study	RFRS classification system	92.59
Lee [4]	Graphical characteristics of BSWFM combined with Euclidean distance	87.4
Tomar and Agarwal [5]	Feature selection-based LSTSVM	85.59
Buscema et al. [6]	TWIST algorithm	84.14
Subbulakshmi et al. [7]	ELM	87.5
Karegowda et al. [8]	GA + Naïve Bayes	85.87
Srinivas et al. [9]	Naïve Bayes	83.70
Polat and Güneş [10]	RBF kernel *F*-score + LS-SVM	83.70
Özşen and Güneş [11]	GA-AWAIS	87.43
Helmy and Rasheed [12]	Algebraic Sigmoid	85.24
Wang et al. [13]	Linear kernel SVM classifiers	83.37
Özşen and Güneş [14]	Hybrid similarity measure	83.95
Kahramanli and Allahverdi [15]	Hybrid neural network method	86.8
Yan et al. [16]	ICA + SVM	83.75
Şahan et al. [17]	AWAIS	82.59
Duch et al. [18]	*K*NN classifier	85.6

BSWFM: bounded sum of weighted fuzzy membership functions; LSTSVM: Least Square Twin Support Vector Machine; TWIST: Training with Input Selection and Testing; ELM: Extreme Learning Machine; GA: genetic algorithm; SVM: support vector machine; ICA: imperialist competitive algorithm; AWAIS: attribute weighted artificial immune system; *K*NN: *k*-nearest neighbor.
